# 1496. Utility of Urinary Lipoarabinomannan (LAM) for Diagnosis of Tuberculosis in HIV-infected Hospitalized Patients – A Prospective Observational Study.

**DOI:** 10.1093/ofid/ofad500.1331

**Published:** 2023-11-27

**Authors:** Deepak Kumar, Shivang Sharma, Gopal Krishana Bohra, M K Garg, Naresh Kumar Midha, Durga Shankar Meena

**Affiliations:** All India Institute of Medical Sciences, Jodhpur (India), Jodhpur, Rajasthan, India; AIIMS, Jodhpur, Rajasthan, India; AIIMS Jodhpur, Jodhpur, Rajasthan, India; AIIMS, Jodhpur, Rajasthan, India; AIIMS, Jodhpur, Rajasthan, India; AIIMS, Jodhpur, Rajasthan, India

## Abstract

**Background:**

Lipoarabinomannan (LAM) is an essential component of the cell wall of mycobacterium tuberculosis. LAM causes anti-inflammatory effects on the human immune system by reducing innate and acquired cellular activity and increasing IL-10 production. Increased LAM in body fluid is a marker of active infection, with maximum concentration in sputum followed by urine. WHO has endorsed the urine LAM for diagnosis of tuberculosis in HIV infection. The study aimed to find the utility of urinary LAM assay in diagnosing tuberculosis in hospitalized patients with HIV Infection.

**Figure:**
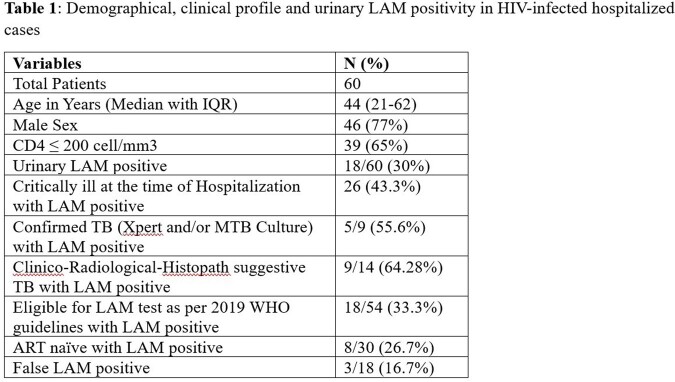

**Methods:**

All Adult (≥18-year-old) patients with HIV infection and hospitalised and regardless of their presenting symptoms or CD4 count, were included for urinary LAM assay (Alere) for diagnosis of tuberculosis. Patients who had taken antituberculosis drugs (first or second line) the month prior were excluded. With molecular and culture methods, best practices were done to find opportunistic infections, including tuberculosis.

**Results:**

A total of 60 patients were enrolled, and urinary LAM was positive in 18 (30%) cases. Among culture or GeneXpert-confirmed tuberculosis, urinary LAM was positive in 55.6% of cases. None of the patients who were not eligible for the LAM test as per 2019 WHO guidelines were found positive (Table 1). The sensitivity, specificity, and negative and positive predictive values of urinary LAM were 62.5%, 91.8%, 76.3% and 85.1%, respectively. In-hospital mortality was significantly high in LAM-positive cases (38.8% vs 16.7%; p-value=0.03).

**Conclusion:**

Urinary LAM may greatly help diagnose tuberculosis in Patients with HIV-TB co-infection with critically ill, with low CD4, or presented by multiple system affection with high specificity. LAM positivity was also associated with increased mortality in HIV infection.

**Disclosures:**

**All Authors**: No reported disclosures

